# Neural Mechanisms Influencing Interlimb Coordination during Locomotion in Humans: Presynaptic Modulation of Forearm H-Reflexes during Leg Cycling

**DOI:** 10.1371/journal.pone.0076313

**Published:** 2013-10-18

**Authors:** Tsuyoshi Nakajima, Rinaldo A. Mezzarane, Taryn Klarner, Trevor S. Barss, Sandra R. Hundza, Tomoyoshi Komiyama, E. Paul Zehr

**Affiliations:** 1 Department of Integrative Physiology, Kyorin University School of Medicine, Mitaka, Tokyo, Japan; 2 Rehabilitation Neuroscience Laboratory, University of Victoria, Victoria, Canada; 3 Laboratory of Signal Processing and Motor Control, College of Physical Education, University of Brasília, Brasília, Brazil; 4 Human Discovery Science, International Collaboration on Repair Discoveries (ICORD), Vancouver, Canada; 5 Centre for Biomedical Research, University of Victoria, Victoria, Canada; 6 Motion and Mobility Laboratory, University of Victoria, Victoria, Canada; 7 Division of Sports and Health Science, Chiba University, Chiba, Japan; 8 Division of Medical Sciences, University of Victoria, Victoria, Canada; University of Alberta, Canada

## Abstract

Presynaptic inhibition of transmission between Ia afferent terminals and alpha motoneurons (Ia PSI) is a major control mechanism associated with soleus H-reflex modulation during human locomotion. Rhythmic arm cycling suppresses soleus H-reflex amplitude by increasing segmental Ia PSI. There is a reciprocal organization in the human nervous system such that arm cycling modulates H-reflexes in leg muscles and leg cycling modulates H-reflexes in forearm muscles. However, comparatively little is known about mechanisms subserving the effects from leg to arm. Using a conditioning-test (C-T) stimulation paradigm, the purpose of this study was to test the hypothesis that changes in Ia PSI underlie the modulation of H-reflexes in forearm flexor muscles during leg cycling. Subjects performed leg cycling and static activation while H-reflexes were evoked in forearm flexor muscles. H-reflexes were conditioned with either electrical stimuli to the radial nerve (to increase Ia PSI; C-T interval  = 20 ms) or to the superficial radial (SR) nerve (to reduce Ia PSI; C-T interval  = 37–47 ms). While stationary, H-reflex amplitudes were significantly suppressed by radial nerve conditioning and facilitated by SR nerve conditioning. Leg cycling suppressed H-reflex amplitudes and the amount of this suppression was increased with radial nerve conditioning. SR conditioning stimulation removed the suppression of H-reflex amplitude resulting from leg cycling. Interestingly, these effects and interactions on H-reflex amplitudes were observed with subthreshold conditioning stimulus intensities (radial n., ∼0.6×MT; SR n., ∼ perceptual threshold) that did not have clear post synaptic effects. That is, did not evoke reflexes in the surface EMG of forearm flexor muscles. We conclude that the interaction between leg cycling and somatosensory conditioning of forearm H-reﬂex amplitudes is mediated by modulation of Ia PSI pathways. Overall our results support a conservation of neural control mechanisms between the arms and legs during locomotor behaviors in humans.

## Introduction

Research in human locomotor control indicates that the neuronal coordination between fore- and hindlimbs observed in quadrupedal locomotor systems is likely preserved in arm and leg interactions [1]–[6]. This coordination may be mediated, at least partly, by coupled pattern generating systems regulating arm (forelimb) and leg (hindlimb) motions [4]–[8] as in other animals. One methodology for assessing this coordination in humans is to measure the modulation of segmental reflexes during movement and somatosensory conditioning [2], [3].

Neuronal transmission from Group Ia afferents to alpha motoneurons in the lumbar spinal cord has been investigated with stationary legs during rhythmic arm movement (e.g. arm cycling). Under such circumstances, the amplitude of the Hoffmann (H-) reflex in the soleus muscle is suppressed in humans by presynaptic inhibition at the Ia afferent – alpha motoneuronal synapse (Ia PSI) [9]–[15]. Interestingly, rhythmic leg movement also leads to suppression of H-reflex amplitude in forearm muscles [16], [17]. These results suggest that a reciprocally organized pattern generating system activated by locomotor commands and afferent feedback modulates excitability of H-reflex pathways in muscles remote from the source of movement [3], [6], [15]. Although a change in excitability of reflex pathways during remote rhythmic movement is indicative of the interlimb linkage for human locomotion [3], [6], the neural mechanisms are still not fully understood.

Presynaptic inhibition of Ia afferent transmission to alpha motoneurons in the pathway for the H-reflex arc may be investigated in humans by using conditioning-test (C-T) stimulation paradigms [18]–[20]. During remote rhythmic arm movement, changes in Ia PSI are associated with soleus H-reflex modulation [6], [9]. Although H-reflexes in forearm flexor carpi radialis (FCR) muscle are similarly suppressed by rhythmic leg cycling [16], comparatively little is known about mechanisms subserving effects from leg to arm. In the upper limb, conditioning stimulation of the radial nerve produces a suppression of *flexor carpi radialis* (FCR) H-reflex amplitude by increasing Ia PSI [19]. In contrast, cutaneous nerve (superficial radial nerve, SR) stimulation facilitates FCR H-reflex amplitude by reducing Ia PSI [20]. Interactions between these somatosensory conditioning effects in forearm H-reflexes and leg cycling movement remain unexplored. Based on the working hypothesis that neural control mechanisms are conserved between human lumbar and cervical spinal networks subserving locomotion [6], we hypothesized that modulation of Ia PSI is the mechanism behind the suppression of FCR H-reflexes during leg rhythmic movement. Portions of these findings have been published as a meeting abstract [21].

## Methods

### Subjects

Thirteen healthy male subjects (aged 20–47 y) participated in two experiments conducted in different sessions. Seven of the 13 subjects participated in both experiments. All participants gave informed written consent to participate in a protocol approved by the local Human Research Ethics Committee at the University of Victoria and conducted in accordance with the Declaration of Helsinki (1964).

### General procedures

**Figure 1:**
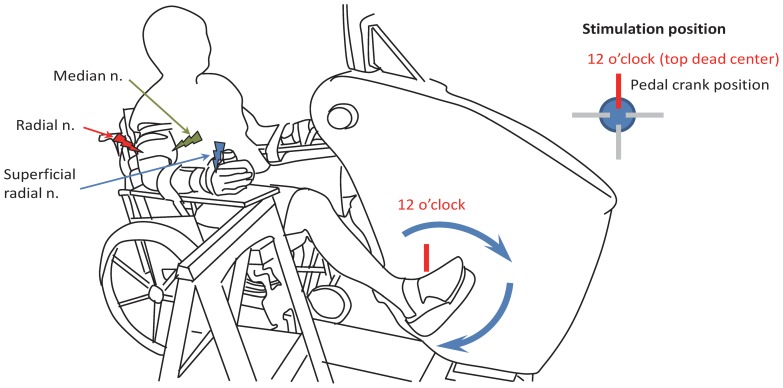
Experimental set-up for leg cycling on the ergometer. Nerve stimulation was delivered during cycling and static trials at the 12'clock position of the right pedal crank (thick lines of red). Green lightning bolt: median nerve stimulation (test stimulation for evoking the H-reflex). Blue lightning bolt: superficial radial nerve stimulation (conditioning stimulation to reduce Ia PSI and facilitate *flexor carpi radialis* (FCR) H-reflex amplitudes) Red lightning bolt: radial nerve stimulation (conditioning stimulation to increase Ia PSI and suppress FCR H-reflex amplitudes).

As shown in Figure 1, subjects performed bilateral leg cycling (∼60 rpm) on an instrumented cycle ergometer (SciFit Pro II SCIFIT Systems, Tulsa UK). The right forearm, wrist and hand were fixed to a rigid platform to minimize any unwanted movement of the arm. A customized brace was also worn to restrict movement and was fixed at the elbow and wrist to preserve angles at ∼120 and 10 degrees, respectively, throughout the experiments. For each trial, subjects maintained a consistent low-level [∼10% of maximal voluntary contraction (MVC)] tonic contraction of their right flexor carpi radialis (FCR) muscle using visual feedback of the rectified and filtered EMG signal which was displayed on a computer screen in real time. Electrical stimulation of median (test stimulation to evoke H-reflexes), radial (conditioning stimulation to increase Ia PSI and reduce H-reflex amplitude) and SR (conditioning stimulation to reduce Ia PSI and increase H-reflex amplitude) nerves was delivered at the 12 o'clock position (top dead center for the right pedal [16], see Figure 1).

### Electrical nerve stimulation

All nerves were stimulated with bipolar electrodes using a Grass S88 stimulator connected in series with SIU5 isolator and CCU1 constant current units (Grass Instruments, Austin, TX, USA). Test (median nerve) and conditioning (radial or SR nerve) electrical stimuli were delivered approximately once every two to three cycles during leg cycling and pseudorandomly between 3 and 5 sec during static trials. Averages of 20 stimuli were calculated for each subject in each condition.

### *Flexor carpi radialis* H-reflexes

FCR H-reflexes in the right arm were evoked by stimulating the median nerve with 1 ms rectangular pulses. Bipolar stimulus electrodes were placed just proximal to the medial epicondyle of the humerus, near the *cubital fossa* [16]. At the start of each experiment, M-wave and H-reflex recruitment curves were constructed from ∼40 responses to determine Mmax and Hmax and to determine stimulus intensities for constant M-wave amplitudes [13]. M-waves were monitored on-line, and stimulation intensity was adjusted as needed to maintain consistent amplitude.

### Somatosensory conditioning to modulate H-reflex amplitudes

The radial nerve was stimulated [0.6–1.0× motor threshold (MT)] with single 1 ms rectangular pulses applied at the spiral groove. MT was defined as the weakest stimulation that produced a motor response in *extensor carpi radialis* (ECR) muscle. During all experiments, subjects maintained a consistent low-level (∼10% of MVC) tonic contraction of FCR muscle using visual feedback as described above. Thus, ECR H-reflexes by stimulating radial nerve were infrequently observed. The interval between the radial nerve conditioning and the test stimulation to evoke an H-reflex (C-T interval) was 20 ms as suggested by previous studies [19], [20].

The cutaneous SR nerve was stimulated with trains of five 1 ms pulses delivered at 300 Hz at 0.5–3 times the radiating threshold (RT). RT was defined as the lowest stimulation intensity required to evoke clear paresthesia throughout the whole innervation territory of the SR nerve [22]. Stimulus electrodes were placed on the dorsal surface of the right forearm just proximal to the radial styloid process. We also measured the minimum stimulation intensity that could be perceived [perceptual threshold (PT)] in all subjects. In all experiments, stimulation of SR nerve was delivered above PT. The C-T interval was 37–47 ms as suggested by previous researchers [20].

### Electromyographic recording

Electromyographic (EMG) activity was recorded from the FCR, ECR, triceps brachii (TB) and soleus (SOL) muscles. EMG signals were obtained with surface electrodes (Thought Technologies LTD., Montreal, Canada) placed in bipolar configuration over the belly of each muscle along the predicted path of the muscle fibres and after reducing skin impedance by light abrasion and alcohol cleaning. All EMG signals were amplified (×1000) and band-pass filtered between 100 Hz and 1 kHz via a bio-amplifier system (P511 Grass Instruments, Austin, TX, USA). All EMG signals were converted to digital data with an analog to digital converter card (National Instruments Corp. TX, USA) and stored on hard disk at a sampling rate of 2 kHz using a custom written computer LabView Program (National Instruments Corp. TX, USA). For all trials, 20 sweeps of data were collected.

### Experimental tasks to manipulate Ia PSI

To explore the presumed presynaptic modulation of the forearm H-reflexes during leg cycling, subjects participated in two experiments. Experiment 1 examined the effect of suprathreshold (i.e. producing an effect in the surface EMG as well as modulation of H-reflex amplitude) conditioning stimulation while Experiment 2 examined subthreshold (i.e. that having no effect detected in the surface EMG) stimulation.

In Experiment 1, we investigated the effects of conditioning nerve stimulation on FCR H-reflex modulation using stimulus intensities that produced significant postsynaptic effects. As defined in previous studies [19], [20] and confirmed in preliminary experiments, we found that conditioning stimulation (radial nerve: 1.0×MT, SR nerve: 3.0×RT) produced short latency suppressive (radial nerve stimulation) and facilitatory (SR nerve stimulation) responses in ongoing FCR EMG. These responses corresponded with the H-reflex duration associated with our C-T intervals [C-T = 20 ms (radial nerve), 37–47 ms (SR nerve)], lasted for ∼40 ms from onset and were evidence of a postsynaptic manifestation of the conditioning stimulation.

The second experiment examined conditioning H-reflex amplitudes using subthreshold intensities. Subthreshold here means stimulation of radial or SR nerves that was insufficient to signficantly modulate ongoing FCR surface EMG in each subject (Experiment 2, n = 9). That is, failed to produce a postsynaptic response detected in the surface EMG. In Experiment 2, therefore, we investigated the effect of somatosensory stimulation (radial and SR nerve conditioning) on H-reflex amplitudes in situations where post-synaptic contributions were minimized. Subthreshold stimulation intensities were defined as those that produced non-signficant reflex EMG responses which were within 2 standard deviations (SD) of the mean prestimulus EMG level. To arrive at these intensities we first confirmed significant (e.g. exceeding 2 SD of mean prestimulus EMG) reflex effects following 1.0×MT for radial (i.e. suppressive responses) and ∼1.0–2.0×RT for SR (i.e. facilitatory responses) stimulation. After this, stimulus intensities were gradually decreased until subthreshold intensities were within 2 SD of the mean rectified EMG. Reflexes following each SR and radial nerve stimulation were compared during static activation and leg cycling. These amplitudes of rectified and averaged ongoing reflex responses were analyzed within the time range that corresponded with the H-reflex duration from onset to offset.

### Data analyses

FCR H-reflex and M-wave amplitudes were averaged for each condition and analyzed off line using Matlab® (Mathworks, Nantick, MA). H-reflex and M-wave amplitudes were normalized to Mmax amplitude. The pre-stimulus EMG activity was calculated as the root mean square value of the EMG signal for 20 ms before stimulation. These EMG amplitudes were normalized to MVC.

Modulation of the FCR H-reflex across motor tasks, amplitudes of H-reflexes, M-waves, and pre-stimulus EMG in the FCR were compared using a two-way repeated measures (RM) ANOVA (2 tasks ×2 stimulus conditions).

Pairwise comparisons were performed on significant main effects (Task and Stimulus condition) and interactions using paired t-tests [23], [24]. The data were expressed as means ± SEM. Significant differences were recognized at *p*<0.05 in all cases. All statistical tests were performed using SPSS software Ver. 11 (SPSS, Chicago, USA).

## Results

### Effect of radial nerve conditioning on the FCR H-reflex during leg cycling

**Figure 2:**
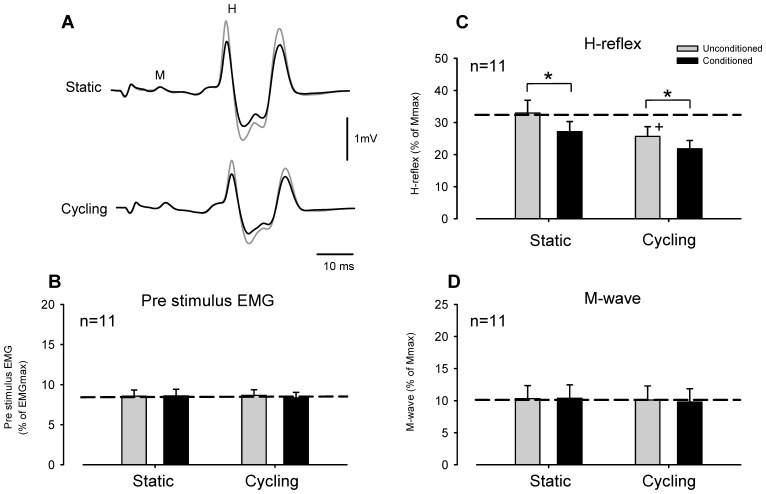
Effect of conditioning the *flexor carpi radialis* H-reflex with radial nerve stimulation during leg cycling and static activation. (A) Typical averaged recordings of conditioned (black lines) and unconditioned (gray lines) H-reflex waveforms during static (upper traces) and cycling (lower traces) tasks obtained from a single subject. Grand means and SEM of magnitudes of the pre-stimulus EMG (B), H-reflex (C) and M-wave (D) during conditioned (black bars) and unconditioned (gray bars) trials. *p<0.01 significantly different from the unconditioned values for each task. +*p*<0.01 significantly different from the unconditioned static value.

Figure 2A shows representative recordings of FCR H-reflex amplitudes from a single subject during radial nerve conditioning (stimulation intensity = 1.0×MT) with static positioning and leg cycling. During static trials without leg movement, suppression of the H-reflex amplitude induced by radial nerve stimulation can be seen. During leg cycling FCR H-reflex amplitude (unconditioned H-reflex) was also reduced, compared with that during the static task, and the amount of suppression was increased by radial nerve conditioning (Fig. 2A, lower traces).

Figures 2B, C and D illustrate pooled data from 11 subjects for the amplitudes of the pre-stimulus EMG activities, H-reflexes and M-waves, respectively, in the FCR muscle. Mean amplitudes of the FCR H-reflex (unconditioned) during leg cycling were significantly smaller than those in the static condition [Fig. 2C, *p*<0.001, unconditioned value; static: 33.0±4.0%, cycling: 25.7±3.0% of Mmax (mean ± SEM)]. Additionally, conditioned FCR H-reflex amplitudes were significantly reduced, compared with unconditioned H-reflex amplitudes during both static and cycling tasks (Fig. 2C, *p*<0.001, conditioned value; static: 27.2±3.2%, cycling: 21.8±2.6%). The two-way RM ANOVA for H-reflex data showed a significant main effect (Task: F^(1,10)^ = 24.641, *p*<0.001, Stimulus condition: F^(1,10)^ = 32.941, *p*<0.001, Task × Stimulus condition: F^(1,10)^ = 3.435 *p*>0.05). M-wave and pre-stimulus EMG data did not significantly differ across stimulus conditions and tasks (Figs. 2B and D; two-way RM ANOVA; M-wave: Task: F^(1,10)^ = 0.577, *p*>0.05, Stimulus condition: F^(1,10)^ = 0.790, *p*>0.05, Task × Stimulus condition: F^(1,10)^ = 1.320, *p*>0.05, pre-stimulus EMG: Task: F^(1,10)^ = 0.042, p>0.05, Stimulus condition: F^(1,10)^ = 0.907, *p*>0.05, Task × Stimulus condition: F^(1,10)^ = 1.619, *p*>0.05).

### Effect of SR nerve conditioning on the FCR H-reflex during leg cycling

**Figure 3:**
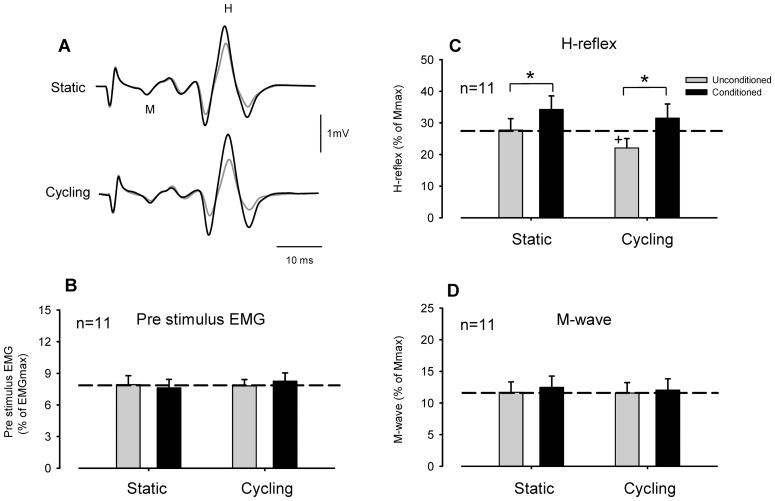
Effect of conditioning the *flexor carpi radialis* H-reflex with superficial radial nerve stimulation during leg cycling and static activation obtained from 11 subjects. Format and abbreviations as in Figure 2.

Figure 3A shows typical recordings of the FCR H-reflex conditioned by SR stimulation with static activation and during leg cycling obtained from a single subject. In the static condition, SR stimulation facilitated FCR H-reflex amplitude. During leg cycling, unconditioned FCR H-reflex amplitudes were reduced but reflex amplitudes with SR conditioning stimulation were larger than those without conditioning stimulation.

These general features were also clear in the group data. Figures 3B, C and D illustrate pooled data for the amplitudes of the pre-stimulus EMG, H-reflex and M-wave in the FCR obtained from 11 subjects. Mean amplitudes of the FCR H-reflex (unconditioned) during leg cycling were significantly smaller than those during static conditions [Fig. 3C, *p*<0.01, static: 27.8±3.6%, cycling: 22.1±3.0% of Mmax (mean ± SEM)]. Furthermore, SR conditioning stimulation removed the suppression of H-reflex amplitude produced by leg cycling (Fig. 3C). The conditioned reflex value during static and leg cycling was 34.3±4.3% and 31.5±4.5% (*p*>0.05), respectively. The two-way RM ANOVA for H-reflex data showed a significant main effect (Task: F^(1,10)^ = 20.849, *p*<0.001, Stimulus condition: F^(1,10)^ = 14.418, *p*<0.01, Task × Stimulus condition: F^(1,10)^ = 3.928 *p*>0.05). Amplitudes of the M-wave and pre-stimulus EMG activities did not differ significantly across stimulus conditions and tasks (Figs. 3B and D, two-way RM ANOVA; M-wave: Task: F^(1,10)^ = 0.366, *p*>0.05, Stimulus condition: F^(1,10)^ = 4.670, *p*>0.05, Task × Stimulus condition: F^(1,10)^ = 1.320, *p*>0.05, pre-stimulus EMG: Task: F^(1,10)^ = 0.657, *p*>0.05, Stimulus condition: F^(1,10)^ = 0.078, p>0.05, Task × Stimulus condition: F^(1,10)^ = 1.939, *p*>0.05).

### Effect of subthreshold radial nerve conditioning on FCR H-reflex amplitudes during leg cycling

**Figure 4:**
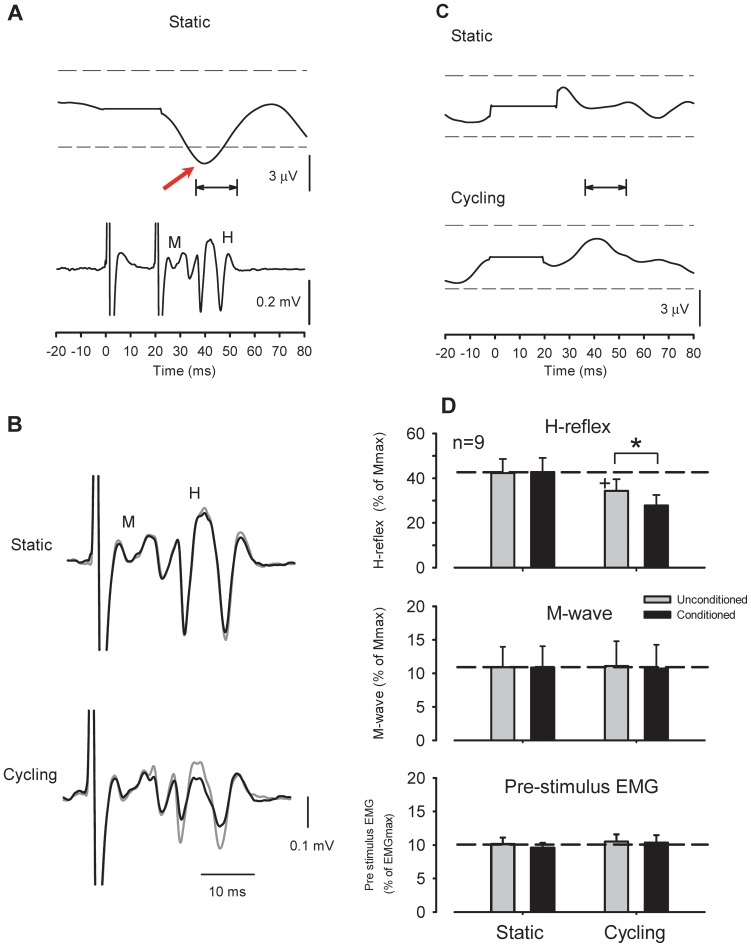
Effect of radial nerve conditioning on *flexor carpi radialis* (FCR) H-reflex amplitudes during leg cycling. (A) Rectified and averaged FCR EMG and H-reflex waveforms following radial nerve stimulation [1.0× motor threshold (MT)] obtained from a single subject. Time zero on the x-axis is onset of conditioning stimulation. Please note that the EMG reflex responses (upper traces) had latencies that corresponded with the H-reflex (lower traces) during the conditioning-test interval. Horizontal arrows show analysis range for assessing ongoing FCR EMG. The arrow shows the suppressive response in the rectified EMG. (B) Conditioning effect of weak radial nerve stimulation (0.6×MT) on FCR H-reflex amplitude during static activation (gray traces) and leg cycling (black traces). (C) EMG responses following weak radial nerve stimulation (0.6×MT) during static and cycling tasks. Non-significant EMG responses were within 2 standard deviations (SD) of the pre-stimulus EMG levels. Broken lines in each panel represent a 2 SD band around the mean pre-stimulus EMG. Note that the stimulus artifact was replaced by the mean of the pre-stimulus EMG. Data in Figures 4A, B, and C were obtained from the same subject. (D) Grand means (± SEM) of H-reflex amplitudes (upper panel), M-waves (middle panel), and pre-stimulus EMG (lower panel) in the FCR muscle during radial nerve conditioning obtained from 9 subjects. * *p*<0.01 significantly different from the unconditioned values for each task. +*p*<0.01 significantly different from the unconditioned static value.

Figure 4A shows representative recordings of the rectified EMG activity and conditioned H-reflex amplitudes following radial nerve stimulation in a single subject. FCR EMG following 1.0× motor threshold stimulation of the radial nerve was suppressed and had a latency that corresponded with the H-reflex evoked with a C-T interval of 20 ms. Therefore, this part of the experiment was designed to test whether the suppression of H-reflex amplitude during leg cycling persisted when applying a weak subthreshold conditioning stimulation that did not significantly modulate the ongoing surface EMG.

Figure 4B depicts the effects of subthreshold radial nerve conditioning on FCR H-reflex amplitudes during static positioning and leg cycling obtained from a single subject (same as Fig. 4A). Radial nerve stimulation at 0.6×MT failed to yield suppressive responses in the rectified and averaged EMG [Fig. 4C, within 2 standard deviations (SD) of mean pre-stimulus EMG]. Conditioning effects (0.6×MT) on the H-reflex amplitude during the static condition were also absent (Fig. 4B, upper traces). During leg cycling, however, H-reflex amplitudes were reduced with this weak (subthreshold for postynaptic effects) conditioning stimulus (Fig. 4B, lower traces). For the group data (n = 9), the amplitudes of the M-wave (Fig. 4D, mid panel) and pre-stimulus EMG activities (Fig. 4D, lower panel) did not differ significantly across stimulus conditions and tasks (two-way RM ANOVA; M-wave: task: F^(1,8)^  = 0.001 *p*>0.05, Stimulus condition: F^(1,8)^  = 0.711, *p*>0.05, Task × Stimulus condition: F^(1,8)^  = 0.011, *p*>0.05, pre-stimulus EMG: Task: F^(1,8)^  = 1.542, *p*>0.05, Stimulus condition: F^(1,8)^  = 0.336, *p*>0.05, Task × Stimulus condition: F^(1,8)^  = 0.642, *p*>0.05).

In addition, there was no significant effect detected in the rectified EMG responses following radial nerve stimulation between static and cycling tasks (radial conditioning: *p* = 0.22, Paired t-test). These responses were within 2 SD of the mean pre-stimulus EMG in all subjects. Importantly, there were no significant differences in conditioning of H-reflex amplitudes during the static condition (*p*>0.05, unconditioned and conditioned value: 42.3±6.4% and 42.8±6.3%, respectively). However H-reflex amplitudes with conditioning stimulation during leg cycling were significantly reduced (Fig. 4D, upper panel, *p*<0.001, unconditioned and conditioned value: 34.3±5.2% and 27.8±4.7%, respectively). The two-way RM ANOVA of H-reflex data showed a significant main effect and interaction (Task: F^(1,8)^  = 25.373, *p*<0.001, Stimulus condition: F^(1,8)^  = 14.938, *p*<0.01, Task × Stimulus condition: F^(1,8)^  = 10.770, *p*<0.01).

### Effect of subthreshold SR nerve conditioning on the FCR H-reflex during leg cycling

In this part of the experiment, we investigated whether the suppression of H-reflex amplitudes during leg cycling could be removed when applying weak, subthreshold SR conditioning stimulation. That is, at an intensity that did not modulate ongoing EMG in the FCR.

**Figure 5:**
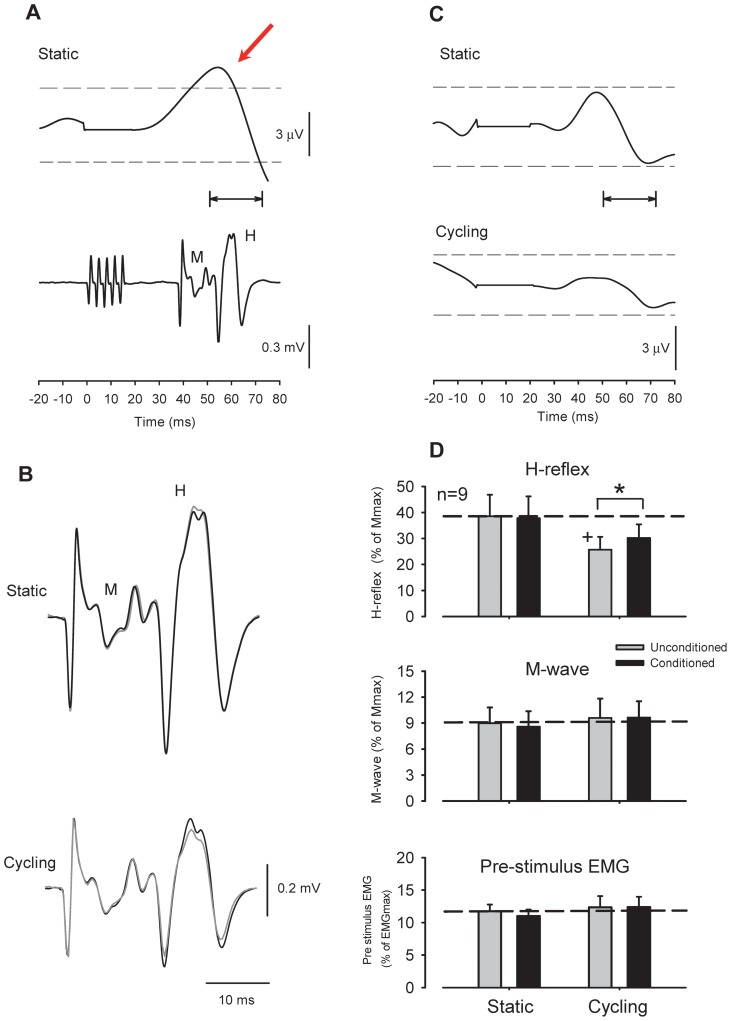
Effect of superficial radial nerve conditioning on *flexor carpi radialis* (FCR) H-reflex amplitudes during leg cycling. (A) Rectified and averaged FCR EMG and H-reflex waveforms following superficial radial (SR) nerve stimulation [1.0× radiating threshold (RT)] obtained from a single subject. (B) Conditioning effect of FCR H-reflex with weak radial nerve stimulation (0.51×RT) during static and cycling tasks. (C) EMG responses following weak SR nerve stimulation (0.51×RT; just above perceptual threshold) during static activation and leg cycling. Please note that figures 5A, B, and C were obtained from same subject. (D) Grand means (± SEM) of H-reflexes (upper panel), M-waves (middle panel), and pre-stimulus EMG levels (lower panel) in the FCR muscle during SR nerve conditioning obtained from 9 subjects. * *p*<0.01 significantly different from the unconditioned values of each tasks. +*p*<0.01 significantly different from the unconditioned static values. Format and abbreviations as in Figure 4.

Figure 5A shows representative recordings of the rectified EMG activities and FCR H-reflex amplitudes following SR nerve stimulation in a single subject. SR nerve stimulation at 1.0×RT still elicited prominant facilitatory responses in FCR EMG at a latency that corresponded with the H-reflex a C-T interval of 37 ms (red arrow in Fig. 5A). During static activiation, when stimulating at 0.51×RT (∼ PT) for the SR nerve, there were no significant effects observed in modulation of H-reflex amplitudes or surface EMG (Fig. 5B and C; below 2 SD of mean pre-stimulus EMG). During leg cycling, however, H-reflexes were facilitated with the weak conditioning stimulation. For the group data (Fig. 5D) obtained from 9 subjects, the amplitudes of the M-wave and pre-stimulus EMG activities did not differ significantly across stimulus conditions and tasks (two-way RM ANOVA; M-wave: Task: F^(1,8)^  = 2.271 *p*>0.05, Stimulus condition: F^(1,8)^  = 0.610, *p*>0.05, Task × Stimulus condition: F^(1,8)^  = 0.763, *p*>0.05, pre-stimulus EMG: Task: F^(1,8)^  = 0.803, *p*>0.05, Stimulus condition: F^(1,8)^  = 0.765, *p*>0.05, Task × Stimulus condition: F^(1,8)^  = 1.666, *p*>0.05). There were no significant differences in rectified EMG amplitudes following conditioning SR nerve between static and cycling tasks (SR conditioning: *p* = 0.23, Paired t-test). These responses were within 2 SD of the mean pre-stimulus EMG in all subjects.

There were no significant differences in the conditioning effects on H-reflex amplitudes during the static condition (*p*>0.05, unconditioned and conditioned value: 38.6±8.2% and 37.9±8.3%, respectively). However, H-reflex amplitudes with SR conditioning during leg cycling were significantly larger than in the unconditioned state (*p*<0.003, unconditioned and conditioned value: 25.7±5.0% and 30.2±5.0%, respectively). In other words, the suppression of H-reflex amplitudes resulting from leg cycling was removed by SR stimulation. The two-way RM ANOVA for H-reflex data showed a significant main effect and interaction (Task: F^(1,8)^  = 7.192, *p*<0.03, Stimulus condition: F^(1,8)^  = 6.764, *p*<0.05, Task × Stimulus condition: F^(1,8)^  = 20.378, p<0.002).

## Discussion

Here we show that leg cycling reduced the H-reflex amplitudes of the forearm flexor muscle by modulation of Ia presynaptic inhibition. There was a clear interaction between modulation of reflex amplitude arising from movement and that from somatosensory conditioning. That is, suppression was increased by an input known to increase Ia PSI (radial nerve conditioning) and was reduced by an input known to reduce Ia PSI (SR conditioning stimulation). These results are consistent with a presynaptic mechanism regulating afferent transmission at the Ia-alpha motoneuronal synapse for arm muscles that is activated by rhythmic leg movement.

### Methodological considerations

The amplitude of the direct M-wave elicited in the FCR was used as an indicator of the stability of the afferent test volley [25], [26]. There were no significant differences in the M-wave amplitudes across all conditions suggesting good stimulus efficacy during the experiments.

Also it is well known that the activation level of the motoneuronal pool influences reflex amplitude [27]. To minimize this effect subjects maintained a constant weak contraction of wrist flexion (∼10% of MVC) across all conditions. Under these situations, pre-stimulus EMG levels of FCR and heteronymous muscles (i.e. ECR and TB) in the arm were well maintained, and there were no significant differences in EMG activities across conditions. Thus, it is unlikely that modulation of H-reflex amplitudes across tasks was the result of simple motoneuronal pool scaling effects such as automatic gain compensation [27].

### Interaction between somatosensory inputs and leg cycling on the suppression of forearm H-reflex amplitudes

Modulation of H-reflexes at similar target muscle activation levels in locomotor tasks is presumed to arise from presynaptic modulation of the Ia afferent volley [25], [28]. A more convincing and direct way to assess presynaptic inhibition of Ia terminals is to use a C-T stimulation paradigm with the H-reflex [9], [18], [19], [29]–[33]. Following stimulation of the radial nerve at motor threshold, suppression of FCR H-reflex amplitudes occur within a C-T interval of ∼5–40 ms [19]. This is within the documented range of C-T intervals for the manifestation of presynaptic inhibition of Ia terminals in the FCR H-reflex pathway (i.e. second phase of inhibition [19]). Berardelli et al. (1987) demonstrated that stimulating the radial nerve with a C-T interval of ∼20 ms elicits the most prominent suppression of H-reflex amplitude in FCR muscle [19]. In contrast, stimulation of the cutaneous SR nerve at C-T intervals of 37–47 ms decreases Ia PSI of the FCR H-reflex pathway leading to facilitation of reflex amplitude [20].

Here we used these C-T intervals and stimulus intensities [19], [20] to investigate whether leg cycling interacted with the effect of radial and SR nerve conditioning. We found suppression of FCR H-reflex amplitudes during leg cycling. This suppression interacted with radial nerve and SR conditioning, inputs known to modulate Ia PSI. We suggest that locomotor control for leg cycling and afferent volleys from somatosensory conditioning stimulation converge on common presynaptic interneurons altering transmission between Ia terminals and alpha motoneurons in the H-reflex pathway [9], [19], [20].

This result parallels an earlier observation of soleus H-reflex modulation during arm cycling [9]. In that study a C-T paradigm involving cutaneous (sural) and antagonist (common peroneal) nerve stimulation was used to determine a presynaptic modulation of soleus H-reflexes activated by rhythmic arm cycling. An important extension of the current work are the data on subthreshold stimulation. This approach was not included in the prior study [9].

### Evidence of presynaptic modulation of the FCR H-reflex amplitude obtained by subthreshold conditioning during leg cycling

From the results in Experiment 1, radial and SR nerves stimulation intensities evoked significant reflexes in the rectified EMG (Figs. 4A and 5B). Thus it is possible that the FCR H-reflex modulation was induced not only by presynaptic mechanisms, but also by postsynaptic effects. In the cat, it has been reported that the effect of relatively short C-T intervals on the monosynaptic reflex pathway overlaps between presynaptic and postsynaptic effects [34]–[36]. Also, motor threshold stimulation of the radial nerve activates other types of afferents (e.g. Group Ib, Group II muscle afferents, and Group II cutaneous afferents) in addition to the larger diameter of Group Ia spindle afferents arising from antagonist muscles [37], [38]. Thus, in Experiment 2 we attempted to investigate modulation of H-reflex using a weak conditioning stimulation (that was subthreshold for postsynaptic effects) during leg cycling.

In Experiment 2, we found that a weak conditioning stimulation (radial nerve: ∼0.6×MT; SR nerve: just above PT) that failed to produce significant responses in the surface ongoing EMG could still elicit modulation of FCR H-reflex amplitudes during leg cycling. The simplest conclusion is that postsynaptic contributions from conditioning volleys were relatively small on the modulation of H-reflex amplitude during leg cycling [33], [35]. Thus, we suggest that the source of FCR H-reflex modulation by conditioning stimulation includes a contribution from Ia presynaptic inhibition activated during leg cycling.

**Figure 6:**
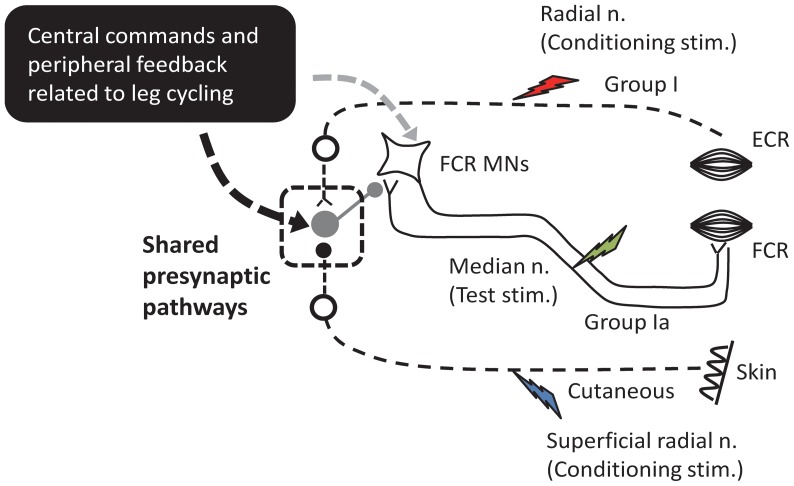
Schematic diagram outlining the possible neural pathways for integration of inputs arising from leg cycling and somatosensory conditioning stimulation on the presumed Ia presynaptic inhibitory pathway. At the center is a simplified H-reflex pathway illustrating group Ia afferents synapsing with alpha motoneurons (MNs) of the *flexor carpi radialis*. Ia PSI and excitability of MNs are regulated by central commands and peripheral feedback related to leg cycling (black square). Inputs from the radial (red lightning bolt) and superficial radial nerves (blue lightning bolt) have excitatory and inhibitory connections onto Ia PSI interneurons (gray circle), respectively. The square with dashed line is a possible shared presynaptic pathways integrating locomotor-related inputs and somatosensory conditioning volleys during locomotion. Green lightning bolt: median nerve stimulation (test stimulation for evoking the FCR H-reflex).

An interesting finding is that the conditioned H-reflex modulation was only observed during the leg cycling task and not during static activation. A schematic representation of the possible circuitry is shown in Figure 6. This figure is an admitted oversimplification of the likely set of connections in the human spinal cord but it can be a useful approximation for discussing our findings and for framing additional research questions. While stationary it is likely that the weak conditioning volleys (thin broken lines) did not reach the threshold for activation of the Ia PSI pathway through the presumed PSI interneurons (large gray circle). Also, the stimulation did not produce reflexes observed in the FCR EMG (an indication of postsynaptic effect by the conditioning stimulation) (see Figs. 4C and 5C). Locomotor inputs play a key role in generating presumed presynaptic modulation of Ia terminals suggesting that the inputs related to leg cycling and conditioning volleys converged onto shared premotoneuronal Ia PSI pathways during leg cycling (see the square with dashed line in Fig. 6). During fictive locomotion in the cat, it has been suggested that afferent and locomotor inputs converge onto shared PSI pathways [39]. This suggests an additional parallel for interlimb locomotor control mechanisms across species.

Recently, Hundza et al. [15] reported that afferent feedback arising from arm cycling is not the primary source responsible for suppression of H-reflex amplitudes in leg muscles. Based on this previous data and assuming a reciprocal organization between nerual control of the arms an legs in humans, it is likely that central commands (e.g. commands for locomotor stepping generators and supraspinal centres) related to leg cycling are key factors in the control mechanism of PSI within the forearm H-reflex pathways [3]. Further studies are needed to evaluate the relative contributions of central and peripheral inputs.

In summary, our data show that presynaptic mechanisms are involved in the modulation of H-reflex amplitude in forearm flexor muscles produced by leg cycling. This extends our prior observations of modulation of H-reflex amplitudes in the lumbar spinal cord (during arm cycling) to the cervical spinal cord. Thus our data support a reciprocal organization between control properties and neural mechanisms modulating reflex excitability in the human spinal cord during locomotor activation.
